# Female Nurses

**Published:** 1861-07

**Authors:** 


FEMALE NURSES.—Thousands of noble-hearted and patriotic women, both North and South, have offered their services, as nurses, during the approaching war. In a number of cities temporary schools have been established for the delivery of practical instruction in this particular art.

